# Strong Linkage Disequilibrium and Proxy Effect of *PPP1R16A* rs109146371 for *DGAT1* K232A in Japanese Holstein Cattle

**DOI:** 10.3390/genes16091000

**Published:** 2025-08-25

**Authors:** Yoshiyuki Akiyama, Takaaki Ando, Nobuhiro Nozaki, Mohammad Arif, Yutaro Ide, Shaohsu Wang, Naoki Miura

**Affiliations:** 1 Joint Graduate School of Veterinary Medicine, Kagoshima University, Kagoshima 890-8580, Japank9236024@kadai.jp (N.M.); 2 Joint Faculty of Veterinary Medicine, Kagoshima University, Kagoshima 890-8580, Japan; 3 Department of Microbiology and Hygiene, Bangladesh Agriculture University, Mymensingh 2202, Bangladesh

**Keywords:** *DGAT1*, GWAS, Holstein cattle, linkage disequilibrium, milk composition, milk yield, *PPP1R16A*, SNP

## Abstract

Background/Objectives: *DGAT1* p. K232A (rs109234250) is a well-established causal variant influencing milk fat and protein content in dairy cattle, but it is often absent from commercial genotyping arrays. *PPP1R16A* rs109146371 frequently appears as a top signal in genome-wide association studies (GWAS) for milk traits. This study aimed to evaluate the linkage disequilibrium (LD) between these two variants in Japanese Holsteins and assess whether rs109146371 exerts an independent effect on milk traits. Methods: A total of 256 Japanese Holstein cows were genotyped for *DGAT1* p. K232A and *PPP1R16A* rs109146371 using TaqMan SNP assays. LD statistics (r^2^, D′) were computed, and linear mixed-effects models were used to evaluate associations with 305-day milk yield, fat percentage, protein percentage, and solids-not-fat (SNF) percentage. Likelihood ratio tests were conducted to assess the independence of SNP effects. Results: Strong LD was observed between *DGAT1* p. K232A and *PPP1R16A* rs109146371 (r^2^ = 0.91, D′ = 0.9962). Both SNPs showed significant associations with all milk production traits; however, model comparisons indicated that rs109146371 did not improve model fit when K232A was included, suggesting no independent effect. Conclusions: *PPP1R16A* rs109146371 serves as a proxy for *DGAT1* K232A rather than an independent determinant of milk traits.

## 1. Introduction

The diacylglycerol O-acyltransferase 1 (*DGAT1*) gene harbors a well-characterized functional polymorphism, p. K232A, which plays a major role in milk fat and protein content in dairy cattle [1,2,3]. A recent meta-analysis further confirmed the strong and consistent association of this variant with milk fat and protein contents across global dairy populations [4]. This polymorphism is caused by a dinucleotide substitution (AA → GC), represented by two adjacent SNPs (rs109234250 and rs109326954), and results in an amino acid change from lysine (K) to alanine (A) in the encoded protein. Following established convention, the p. K allele is considered wild-type, and the p. A allele is treated as a mutant variant. Despite its functional importance, *DGAT1* p. K232A is not directly included in most commercial genotyping arrays, and its effect is often inferred in genome-wide association studies (GWAS) through imputation or linkage with nearby markers.

Two single-nucleotide polymorphisms (SNPs) on BTA14, rs109146371 (Illumina ID: BFGL-NGS-57820; BTA14: 465,742) and rs109421300 (Illumina ID: ARS-BFGL-NGS-4939; BTA14: 609,870), have been identified as top association signals for milk production traits in multiple GWAS [5,6,7,8,9,10,11,12,13,14]. Both markers have been included on the Illumina BovineSNP50 BeadChip since Version 1.0, and are also present on the BovineHD Genotyping BeadChip, leading to their frequent detection in studies using these arrays [5,6,7,8,9,13,14]. They have also been detected in studies using genotypes imputed from multiple commercial SNP chips [10], application of different SNP panels [11] or targeted genotyping using Sequenom MassARRAY [12]. According to these studies, both markers showed significant associations with milk yield, fat yield, fat percentage, protein yield, and protein percentage in multiple cattle populations. rs109146371is commonly annotated as *PPP1R16A* or *FOXH1* and lies upstream of *DGAT1*, whereas rs109421300 is located immediately upstream of *DGAT1* and proximal to the causal K232A variant (BTA14: 611,019). Notably, rs109421300 has been shown to be in near-complete LD with K232A (r^2^ = 0.998), as demonstrated by Wang et al. [15], confirming its role as a well-established proxy marker for *DGAT1*.

In this study, we aimed to evaluate the LD between *DGAT1* p.K232A and *PPP1R16A* rs109146371 in a Japanese Holstein population and to determine whether rs109146371 exerts an independent effect on milk production traits.

## 2. Materials and Methods

### 2.1. Animals and Ethical Statement

The study population consisted of 256 cows from a commercial dairy farm in Hokkaido, Japan. Ear tissue samples were collected as by-products of routine ear tagging in 2019 under Japan’s national cattle identification program using Tissue Sampling Units (Allflex, Dallas, TX, USA). No live animal procedures were performed, and no experimental intervention was applied. Therefore, formal ethical approval was waived in accordance with national guidelines.

### 2.2. Phenotypic Data

Phenotypic data (covering actual 305-day milk yield, mean fat percentage, mean protein percentage, and mean solids-not-fat [SNF] percentage, together with parity information) were retrieved from the national Dairy Herd Improvement (DHI) system. Only cows with complete records for the entire 305-day period had these values available in the system, and these records were used for analysis. The milk yield values represent the cumulative total over the 305-day period, obtained from daily yield records in the farm’s milking system, while the compositional traits represent the average of all measurements recorded during the same period. This approach provides a standardized measure that minimizes short-term fluctuations due to season or stage of lactation and reflects the cow’s overall performance [16]. A total of 632 complete 305-day lactation records from January 2011 to March 2025 were available for the 256 cows included in this study, covering both primiparous and multiparous lactations. Feeding and management practices had been largely uniform for all animals whose data were evaluated in this study. The cows had been fed a total mixed ration (TMR) ad libitum, consisting primarily of grass silage, corn silage, concentrate mix, ground corn, and soybean meal, with only minor year-to-year variation in ingredient proportions.

### 2.3. DNA Extraction and Genotyping

Genomic DNA was extracted from ear tissue samples using the GeneJET Genomic DNA Purification Kit (Thermo Fisher Scientific, Waltham, MA, USA) according to the manufacturer’s protocol. Genotyping was performed with Custom TaqMan SNP Genotyping Assays (Thermo Fisher Scientific, Waltham, MA, USA) on a StepOne Plus Real-Time PCR System (Thermo Fisher Scientific, Waltham, MA, USA), following the manufacturer’s instructions. Two loci were analyzed: *DGAT1* p. K232A (Assay ID: ANH6HP7) and *PPP1R16A* rs109146371 (Assay ID: ANEP2MD).

### 2.4. Data Availability

All phenotype (with parity information) and genotype datasets used in this study are publicly available at Figshare (https://doi.org/10.6084/m9.figshare.29324261) under a CC-BY license. Data can be accessed without restrictions.

### 2.5. Statistical Analysis

Genotype and allele frequencies were calculated, and Hardy–Weinberg equilibrium (HWE) was tested using exact tests. Linkage disequilibrium (LD) between *DGAT1* p. K232A and *PPP1R16A* rs109146371 was evaluated using r^2^ and D′.

Association analysis consisted of two steps. In the first step, each SNP was analyzed separately to estimate least squares means (LSM) and test the main effect of genotype using the following linear mixed-effects model:

(1) $$y_{ijk}=\mu +G_{i}+P_{j}+{\mathrm{cow}}_{k}+e_{ijk}$$

where $y_{ijk}$ is the phenotypic record (305-day milk yield, fat %, protein %, or SNF %),

μ is the overall mean,
$G_{i}$ is the fixed effect of SNP genotype,
$P_{j}$ is the fixed effect of parity,
${\mathrm{cow}}_{k}$ is the random effect of the cow,
and $e_{ijk}$ is the residual error.

Models were fitted by restricted maximum likelihood (REML), and pairwise comparisons of LSM were performed using Tukey’s HSD test.

In the second step, to evaluate whether rs109146371 explained additional variance beyond p. K232A, two nested models were compared using likelihood ratio tests (LRT) based on maximum likelihood (ML) estimation:

Model 1:

(2) $$y_{ijk}=\mu +G_{1i}+P_{j}+{\mathrm{cow}}_{k}+e_{ijk},$$

and Model 2:

(3) $$y_{ijk}=\mu +G_{1i}+G_{2l}+P_{j}+{\mathrm{cow}}_{k}+e_{ijk}$$

Model fit was assessed using the Akaike Information Criterion (AIC), the Bayesian Information Criterion (BIC), and log-likelihood. All analyses were conducted in R version 4.4.3 [17] using its HardyWeinberg, genetics, lme4, lmerTest, and emmeans packages [18,19,20,21,22].

Descriptive statistics for raw phenotypic data are presented as mean ± standard deviation (SD) because SD reflects the variability among individual animals in the sample, whereas standard error (SE) reflects the uncertainty in estimating the population mean. Since our aim was to describe the observed distribution of raw phenotypic data, we used SD in the present study [23].

Generative AI Usage: AI-assisted tools (ChatGPT-4o) were used only for language refinement and formatting guidance; no AI was used for data generation, analysis, or interpretation.

## 3. Results

### 3.1. Genotype and Allele Frequencies

Genotype and allele frequencies for *DGAT1* p. K232A and *PPP1R16A* rs109146371 were calculated in 256 Japanese Holstein cows. Both loci were in Hardy–Weinberg equilibrium (*p* = 0.374 for *DGAT1*; *p* = 0.542 for *PPP1R16A*). The most frequent genotypes were AA for *DGAT1* (56.2%) and TT for *PPP1R16A* (53.9%), and allele A and T were predominant at these loci (Table 1).

### 3.2. Linkage Disequilibrium Between DGAT1 and PPP1R16A

Genotype cross-tabulation showed a restricted pattern, with only five of nine possible genotype combinations observed. All KK genotypes co-occurred with CC, and all TT genotypes co-occurred with AA (Table 2). Despite the physical distance of approximately 145 kb, LD was extremely strong (r^2^ = 0.91; D′ = 0.9962).

### 3.3. Descriptive Statistics for Milk Traits

Descriptive statistics for the analyzed milk traits are summarized in Table 3. The mean 305-day milk yield was 10,350.54 kg (range: 4895.5–15,938.1), with fat and protein percentages averaging 3.78% and 3.33%, respectively. SNF percentage had a mean of 8.84% and ranged from 7.94% to 9.69%. The average parity was 2.63, with cows ranging from first to ninth lactation.

### 3.4. Association Analysis with Milk Traits

Both *DGAT1* p. K232A and *PPP1R16A* rs109146371 were significantly associated with 305-day milk yield, fat percentage, protein percentage, and SNF percentage (*p* < 0.001). The patterns of effect for *PPP1R16A* mirrored those of *DGAT1*, with milk yield increasing and fat decreasing from KK/CC to AA/TT (Table 4). Supplementary Table S1 provides least squares means, standard errors, 95% confidence intervals, and ANOVA *p*-values for each trait–SNP combination. Supplementary Table S2 presents Tukey HSD and *t*-test *p*-values for all pairwise genotype comparisons.

### 3.5. Model Comparisons for Independent Effects

Based on likelihood ratio tests, we evaluated whether rs109146371 explained additional variance beyond *DGAT1*. Across all traits, inclusion of rs109146371 did not significantly improve model fit (Table 5), indicating its effect is largely due to LD with *DGAT1*.

## 4. Discussion

### 4.1. Strong LD Between DGAT1 K232A and PPP1R16A rs109146371

Since its discovery more than 20 years ago [1], the *DGAT1* p. K232A polymorphism has remained a major focus of dairy cattle genetics research. Even in recent years, investigations have continued across various breeds and geographic regions, consistently confirming its strong association with milk composition traits under diverse genetic backgrounds [24,25,26]. Numerous studies have also examined its impact under different management and nutritional conditions. For example, Tumino et al. [27] reported that the magnitude of the K allele’s effect on milk fat composition, particularly on short- and medium-chain fatty acids, was greater in pasture-based systems, likely due to higher ruminal production of acetic acid, a key substrate for de novo fatty acid synthesis, compared with indoor TMR-fed systems. Similarly, Matamoros et al. [28] found that acetate supplementation increased milk fat concentration and yield regardless of *DGAT1* genotype or parity, while short-term responses suggested a possible genotype-dependent effect. In parallel, functional genomics approaches have been employed to explore the molecular mechanisms underlying this polymorphism, such as modulation of gene expression through multi-junction exon splice enhancement [29] and its influence on pre-mRNA splicing efficiency [30]. Collectively, these findings highlight the enduring importance of p. K232A, not only as a well-established causal mutation for milk composition traits but also as a model for studying gene—environment interactions and gene function in livestock. In the present study, the observed LD between these two loci was exceptionally strong, with r^2^ = 0.91 and D′ = 0.996. This extended LD is likely explained by the genomic architecture of this region and accounts for the frequent detection of rs109146371 as a significant association signal in GWAS, despite its location approximately 145 kb upstream and annotation to a different gene. These results indicate that this repeated detection does not represent a novel causal variant but rather reflects the effect of K232A.

### 4.2. Implications for GWAS Interpretation and Genomic Selection

These findings clarify why association signals near *PPP1R16A* frequently appear in GWAS of milk traits. *DGAT1* p. K232A is absent from most commercial SNP arrays, and its effect is often inferred from nearby markers. Previous research showed that rs109421300 (ARS-BFGL-NGS-4939), located immediately upstream of *DGAT1*, is in near-complete LD with K232A (r^2^ = 0.998) [15]. Our results indicate that *PPP1R16A* rs109146371 behaves similarly, showing strong association signals due to LD rather than an independent effect. This explains its frequent detection in GWAS and suggests that such signals should be interpreted in the context of the strong LD block surrounding *DGAT1*.

### 4.3. Limitations and Future Directions

A key limitation of this study is the relatively small sample size (*n* = 256) and the fact that all animals were derived from a single herd. As a result, the observed patterns may not fully represent the haplotype diversity present in the broader Japanese or global Holstein populations. To confirm the generalizability of these findings, further validation using large-scale, multi-herd datasets and whole-genome sequencing (WGS) will be necessary. In addition, it remains unclear whether the strong LD between *DGAT1* K232A and rs109146371 observed in Holsteins can be extrapolated to other dairy breeds.

While our results indicate that rs109146371 functions as a proxy for *DGAT1* K232A, future functional studies targeting regulatory regions near *PPP1R16A* may help determine whether this locus has any biological role beyond its linkage with *DGAT1*.

All animals were from a single herd subject to largely uniform management practices, which minimizes environmental variation versus that seen in multi-herd datasets. However, we cannot completely exclude some degree of phenotypic variation due to year-to-year changes in feeding or other management practices. The use of actual 305-day milk yield and mean composition, along with parity adjustment in the models, helps reduce seasonal and stage-of-lactation effects and ensures a more robust phenotypic measure.

## 5. Conclusions

In conclusion, our study demonstrated that *PPP1R16A* rs109146371 is strongly linked to *DGAT1* p. K232A and does not provide an independent genetic signal for milk production traits in Japanese Holstein cattle. These results confirm that the *PPP1R16A* association observed in GWAS is LD-driven and highlight the importance of distinguishing true causal variants from proxy markers when interpreting association signals. This insight is important for genomic selection strategies, as it helps prevent misinterpretation of proxy markers as independent targets.

## Figures and Tables

**Table 1:** Genotype and allele frequencies for *DGAT1* p. K232A and *PPP1R16A* rs109146371, with HWE *p*-values.

		Genotypes		Alleles		*p*-Value
		*n*	Frequency (%)	*n*	Frequency (%)	
	KK (K)	12	4.7	124	24.2	
*DGAT1* p. K232A	KA	100	39.1			0.374
	AA (A)	144	56.2	388	75.8	
	CC (C)	15	5.9	133	26.0	
*PPP1R16A*	CT	103	40.2			0.542
rs109146371	TT(T)	138	53.9	379	74.0	

Note: Genotype frequency is shown as both count (*n*) and percentages. Allele frequencies were calculated based on observed genotype counts. *p*-values were obtained using exact tests to assess deviation from HWE.

**Table 2:** Genotype cross-tabulation and LD statistics between *DGAT1* p. K232A and *PPP1R16A* rs109146371.

		*PPP1R16A* rs109146371			LD Test	
*DGAT1* p. K232A		CC	CT	TT	r^2^	D′
	KK	12	0	0		
	KA	3	97	0	0.91	0.9962
	AA	0	6	138		

**Table 3:** Descriptive statistics (mean ± SD and range) for parity and milk production traits.

Parity and Milk Traits	Mean ± SD (Min–Max)
Parity	2.63 ± 1.54 (1–9)
305-day Milk Yield (kg)	10,350.54 ± 1760.15 (4895.5–15,938.1)
Fat percentage	3.78 ± 0.38 (2.63–5.07)
Protein percentage	3.33 ± 0.21 (2.72–4.1)
SNF percentage	8.84 ± 0.26 (7.94–9.69)

**Table 4:** LSM (±SE) of 305-day milk yield, fat %, protein %, and SNF % by SNP genotypes.

		LSM ± SE			
SNP	Genotype	305-day Milk Yield (kg)	Fat (%)	Protein (%)	SNF (%)
	KK	9753.96 ^b^ ± 381.72	4.27 ^a^ ± 0.08	3.39 ^a^ ± 0.05	8.87 ^a^ ± 0.06
*DGAT1*	KA	10346.24 ^b^ ± 186.27	3.96 ^b^ ± 0.04	3.36 ^b^ ± 0.02	8.82 ^b^ ± 0.03
p. K232A	AA	10842.49 ^a^ ± 175.62	3.57 ^c^ ± 0.04	3.19 ^b^ ± 0.02	8.64 ^b^ ± 0.03
	*p*-value	*p* < 0.001 ***	*p* < 0.001 ***	*p* < 0.001 ***	*p* < 0.001 ***
		LSM ± SE			
SNP	Genotype	305-day Milk Yield (kg)	Fat (%)	Protein (%)	SNF (%)
	CC	9695.84 ^b^ ± 350.56	4.24 ^a^ ± 0.08	3.38 ^a^ ± 0.05	8.84 ^a^ ± 0.06
*PPP1R16A*	CT	10374.97 ^b^ ± 185.3	3.93 ^b^ ± 0.04	3.34 ^a^ ± 0.02	8.81 ^a^ ± 0.03
rs109146371	TT	10846.4 ^a^ ± 176.18	3.57 ^c^ ± 0.04	3.2 ^b^ ± 0.02	8.64 ^b^ ± 0.03
	*p*-value	*p* < 0.001 ***	*p* < 0.001 ***	*p* < 0.001 ***	*p* < 0.001 ***

Note: *p*-values shown are from ANOVA for the main effect of genotype; Significance levels: *** *p* < 0.001; Different superscript letters (a, b, c) indicate significant differences among genotypes based on Tukey’s HSD test (*p* < 0.05).

**Table 5:** Model comparison using likelihood ratio tests (LRT) for inclusion of *PPP1R16A* after *DGAT1*.

Milk Traits	Model	AIC	BIC	logLik	LRT (χ^2^)	df	*p*-Value
305-day milk yield	*DGAT1* only	10,928	10,985.8	−5451			
	*DGAT1* + *PPP1R16A*	10,930.3	10,997	−5450.1	1.71	2	0.425
Fat percentage	*DGAT1* only	183.1	240.9	−78.5			
	*DGAT1* + *PPP1R16A*	186.1	252.8	−78	0.96	2	0.618
Protein percentage	*DGAT1* only	−552.3	−494.4	289.1			
	*DGAT1* + *PPP1R16A*	−552.1	−485.3	291	3.79	2	0.15
SNF percentage	*DGAT1* only	−294.2	−236.3	160.1			
	*DGAT1* + *PPP1R16A*	−293.3	−226.5	161.6	3.1	2	0.212

Note: The LRT was used to assess whether the inclusion of *PPP1R16A* significantly improved model fit. Degrees of freedom (df) and *p*-values are based on the difference in model deviance.

## Data Availability

The datasets analyzed during the current study are publicly available at Figshare: https://doi.org/10.6084/m9.figshare.29324261.

## Supplementary Materials

The following supporting information can be downloaded at: https://www.mdpi.com/article/10.3390/genes16091000/s1, Table S1: Least squares means (LSMs) and ANOVA *p*-values for genotype effects of DGAT1 p.K232A and PPP1R16A rs109146371 on 305-day milk yield and composition traits.; Table S2: Pairwise comparison *p*-values for each trait–SNP combination.

## Abbreviations

The following abbreviations are used in this manuscript:

LD	Linkage disequilibrium
SNP	Single nucleotide polymorphism
GWAS	Genome-wide association study
DHI	Dairy Herd Improvement
TMR	Total mixed ration
LSM	Least squares mean
HWE	Hardy–Weinberg equilibrium
AIC	Akaike Information Criterion
BIC	Bayesian Information Criterion
WGS	Whole-genome sequencing

## References

[1] Grisart B., Coppieters W., Farnir F., Karim L., Ford C., Berzi P., Cambisano N., Mni M., Reid S., Simon P. (2002). Positional candidate cloning of a QTL in dairy cattle: Identification of a missense mutation in the bovine *DGAT1* gene with major effect on milk yield and composition. Genome Res..

[2] Grisart B., Farnir F., Karim L., Cambisano N., Kim J.J., Kvasz A., Mni M., Simon P., Frere J.M., Coppieters W. (2004). Genetic and functional confirmation of the causality of the *DGAT1* K232A quantitative trait nucleotide in affecting milk yield and composition. Proc. Natl. Acad. Sci. USA.

[3] Winter A., Kramer W., Werner F.A., Kollers S., Kata S., Durstewitz G., Buitkamp J., Womack J.E., Thaller G., Fries R. (2002). Association of a lysine-232/alanine polymorphism in a bovine gene encoding acyl-CoA:diacylglycerol acyltransferase (*DGAT1*) with variation at a quantitative trait locus for milk fat content. Proc. Natl. Acad. Sci. USA.

[4] Mahmoudi P., Rashidi A. (2023). Strong evidence for association between K232A polymorphism of the *DGAT1* gene and milk fat and protein contents: A meta-analysis. J. Dairy Sci..

[5] Buitenhuis B., Janss L.L., Poulsen N.A., Larsen L.B., Larsen M.K., Sørensen P. (2014). Genome-wide association and biological pathway analysis for milk-fat composition in Danish Holstein and Danish Jersey cattle. BMC Genom..

[6] Cole J.B., Wiggans G.R., Ma L., Sonstegard T.S., Lawlor T.J., Crooker B.A., Van Tassell C.P., Yang J., Wang S., Matukumalli L.K. (2011). Genome-wide association analysis of thirty one production, health, reproduction and body conformation traits in contemporary U.S. Holstein cows. BMC Genom..

[7] Capomaccio S., Milanesi M., Bomba L., Cappelli K., Nicolazzi E.L., Williams J.L., Ajmone-Marsan P., Stefanon B. (2015). Searching new signals for production traits through gene-based association analysis in three Italian cattle breeds. Anim. Genet..

[8] Nayeri S., Sargolzaei M., Abo-Ismail M.K., May N., Miller S.P., Schenkel F., Moore S.S., Stothard P. (2016). Genome-wide association for milk production and female fertility traits in Canadian dairy Holstein cattle. BMC Genet..

[9] Jiang L., Liu J., Sun D., Ma P., Ding X., Yu Y., Zhang Q. (2010). Genome Wide Association Studies for Milk Production Traits in Chinese Holstein Population. PLoS ONE.

[10] Jiang J., Ma L., Prakapenka D., Vanraden P.M., Cole J.B., Da Y. (2019). A Large-Scale Genome-Wide Association Study in U.S. Holstein Cattle. Front. Genet..

[11] Wang T., Li J., Gao X., Song W., Chen C., Yao D., Ma J., Xu L., Ma Y. (2020). Genome-wide association study of milk components in Chinese Holstein cows using single nucleotide polymorphism. Livest. Sci..

[12] Yang Z., Lian Z., Liu G., Deng M., Sun B., Guo Y., Liu D., Li Y. (2021). Identification of genetic markers associated with milk production traits in Chinese Holstein cattle based on post genome-wide association studies. Anim. Biotechnol..

[13] Bohlouli M., Halli K., Yin T., Gengler N., König S. (2022). Genome-wide associations for heat stress response suggest potential candidate genes underlying milk fatty acid composition in dairy cattle. J. Dairy Sci..

[14] Cortes-Hernández J.G., García-Ruiz A., Peñagaricano F., Montaldo H.H., Ruiz-López F.J. (2025). Uncovering the genetic basis of milk production traits in Mexican Holstein cattle based on individual markers and genomic windows. PLoS ONE.

[15] Wang X., Wurmser C., Pausch H., Jung S., Reinhardt F., Tetens J., Thaller G., Fries R. (2012). Identification and Dissection of Four Major QTL Affecting Milk Fat Content in the German Holstein-Friesian Population. PLoS ONE.

[16] Leblanc S. (2010). Assessing the Association of the Level of Milk Production with Reproductive Performance in Dairy Cattle. J. Reprod. Dev..

[17] R Core Team (2025). R: A Language and Environment for Statistical Computing.

[18] Graffelman J. (2015). Exploring Diallelic Genetic Markers: TheHardyWeinbergPackage. J. Stat. Softw..

[19] Warnes G.R., Bolker B., Lumley T. (2022). *Genetics: Population Genetics*; R package version 1.3.8.1.2. https://CRAN.R-project.org/package=genetics.

[20] Bates D., Mächler M., Bolker B., Walker S. (2015). Fitting Linear Mixed-Effects Models Usinglme4. J. Stat. Softw..

[21] Kuznetsova A., Brockhoff P.B., Christensen R.H.B. (2017). lmerTest Package: Tests in Linear Mixed Effects Models. J. Stat. Softw..

[22] Lenth R.V. (2023). *Emmeans: Estimated Marginal Means, Aka Least-Squares Means*; R package version 1.8.8. https://CRAN.R-project.org/package=emmeans.

[23] Barde P., Barde M. (2012). What to use to express the variability of data: Standard deviation or standard error of mean?. Perspect. Clin. Res..

[24] Wang Q., Bovenhuis H. (2020). Combined use of milk infrared spectra and genotypes can improve prediction of milk fat composition. J. Dairy Sci..

[25] Gothwal A., Magotra A., Bangar Y.C., Malik B.S., Yadav A.S., Garg A.R. (2023). Candidate K232A mutation of *DGAT1* gene associated with production and reproduction traits in Indian Dairy cattle. Anim. Biotechnol..

[26] Elzaki S., Korkuć P., Arends D., Reissmann M., Brockmann G.A. (2022). Effects of *DGAT1* on milk performance in Sudanese Butana × Holstein crossbred cattle. Trop. Anim. Health Prod..

[27] Tumino S., Criscione A., Moltisanti V., Marletta D., Bordonaro S., Avondo M., Valenti B. (2021). Feeding System Resizes the Effects of *DGAT1* Polymorphism on Milk Traits and Fatty Acids Composition in Modicana Cows. Animals.

[28] Matamoros C., Dechow C.D., Harvatine K.J. (2023). Interaction of *DGAT1* polymorphism, parity, and acetate supplementation on feeding behavior, milk synthesis, and plasma metabolites. J. Dairy Sci..

[29] Fink T., Lopdell T.J., Tiplady K., Handley R., Johnson T.J.J., Spelman R.J., Davis S.R., Snell R.G., Littlejohn M.D. (2020). A new mechanism for a familiar mutation-bovine *DGAT1* K232A modulates gene expression through multi-junction exon splice enhancement. BMC Genom..

[30] Gaiani N., Bourgeois-Brunel L., Rocha D., Boulling A. (2023). Analysis of the impact of *DGAT1* p.M435L and p.K232A variants on pre-mRNA splicing in a full-length gene assay. Sci. Rep..

