# Supplementary material for: Strong Linkage Disequilibrium and Proxy Effect of PPP1R16A rs109146371 for DGAT1 K232A in Japanese Holstein Cattle
Source: Genes (Basel). 2025 Aug 25;16(9):1000. doi: 10.3390/genes16091000 (PMC12469489; doi:10.3390/genes16091000)
Supplement: Supplementary file 1 [file genes-16-01000-s001.zip › genes-3823933-supplementary.pdf]

Supplementary Materials

Table S1. Least squares means and ANOVA *p*-values for each trait–SNP combination.

Variable	SNP	Genotype	LSM	SE	LCL	UCL	*p*-value
305-day Milk Yield (kg)	*DGAT1* p.K232A	KK	9753.959	381.721305	9003	10504.92	0.000349399
305-day Milk Yield (kg)	*DGAT1* p.K232A	KA	10346.24	186.271756	9980.34	10712.13	
305-day Milk Yield (kg)	*DGAT1* p.K232A	AA	10842.49	175.616979	10497.58	11187.4	
305-day Milk Yield (kg)	*PPP1R16A* rs109146371	CC	9695.84	350.56336	9006.334	10385.35	1.76E-04
305-day Milk Yield (kg)	*PPP1R16A* rs109146371	CT	10374.97	185.304674	10010.98	10738.96	
305-day Milk Yield (kg)	*PPP1R16A* rs109146371	TT	10846.4	176.176005	10500.39	11192.41	
305-day average Fat %	*DGAT1* p.K232A	KK	4.270855	0.08455408	4.104499	4.437212	2.80E-28
305-day average Fat %	*DGAT1* p.K232A	KA	3.957578	0.03907485	3.880823	4.034332	
305-day average Fat %	*DGAT1* p.K232A	AA	3.573251	0.03631021	3.50194	3.644562	
305-day average Fat %	*PPP1R16A* rs109146371	CC	4.242364	0.07858264	4.087789	4.396938	4.43E-26
305-day average Fat %	*PPP1R16A* rs109146371	CT	3.933798	0.03931366	3.856575	4.01102	
305-day average Fat %	*PPP1R16A* rs109146371	TT	3.574943	0.03691003	3.502453	3.647433	
305-day average Protein %	*DGAT1* p.K232A	KK	3.390339	0.05179948	3.288411	3.492268	5.09E-13
305-day average Protein %	*DGAT1* p.K232A	KA	3.3597	0.02263514	3.315233	3.404167	
305-day average Protein %	*DGAT1* p.K232A	AA	3.194221	0.02070045	3.153565	3.234878	
305-day average Protein %	*PPP1R16A* rs109146371	CC	3.382563	0.04807438	3.287983	3.477144	2.70E-10
305-day average Protein %	*PPP1R16A* rs109146371	CT	3.34391	0.02280775	3.299105	3.388716	
305-day average Protein %	*PPP1R16A* rs109146371	TT	3.199624	0.02114105	3.158101	3.241147	
305-day average SNF %	*DGAT1* p.K232A	KK	8.869878	0.06452243	8.742911	8.996846	5.12E-11
305-day average SNF %	*DGAT1* p.K232A	KA	8.822549	0.02794689	8.767646	8.877452	
305-day average SNF %	*DGAT1* p.K232A	AA	8.636912	0.02549003	8.586848	8.686976	
305-day average SNF %	*PPP1R16A* rs109146371	CC	8.840843	0.05960688	8.72357	8.958116	7.93E-09
305-day average SNF %	*PPP1R16A* rs109146371	CT	8.808036	0.02807634	8.752879	8.863193	
305-day average SNF %	*PPP1R16A* rs109146371	TT	8.642167	0.02597729	8.591144	8.69319	

Note: Least squares means (LSMs), standard errors (SE), 95% confidence intervals (LCL–UCL), and the ANOVA *p*-values for genotype main effects are shown for each candidate SNP and 305-day variable.

Table S2. Pairwise comparison *p*-values for each trait–SNP combination.

Variable	SNP	contrast	*Tukey HSD p*-value	*t-test p*-value
305-day Milk Yield (kg)	*DGAT1* p.K232A	AA - KA	0.004111437	0.001444543
305-day Milk Yield (kg)	*DGAT1* p.K232A	AA - KK	0.008484743	0.003029107
305-day Milk Yield (kg)	*DGAT1* p.K232A	KA - KK	0.24504146	0.109565649
305-day Milk Yield (kg)	*PPP1R16A* rs109146371	CC - CT	1.09E-01	0.044261461
305-day Milk Yield (kg)	*PPP1R16A* rs109146371	CC - TT	0.001740789	0.000602932
305-day Milk Yield (kg)	*PPP1R16A* rs109146371	CT - TT	0.006814798	0.002420337
305-day average Fat %	*DGAT1* p.K232A	AA - KA	0.00E+00	4.8532E-23
305-day average Fat %	*DGAT1* p.K232A	AA - KK	2.19824E-14	1.67423E-15
305-day average Fat %	*DGAT1* p.K232A	KA - KK	0.000626699	0.000214575
305-day average Fat %	*PPP1R16A* rs109146371	CC - CT	2.54E-04	8.63E-05
305-day average Fat %	*PPP1R16A* rs109146371	CC - TT	1.10E-13	2.50E-16
305-day average Fat %	*PPP1R16A* rs109146371	CT - TT	1.64E-13	3.84E-20
305-day average Protein %	*DGAT1* p.K232A	AA - KA	2.70E-12	8.63342E-13
305-day average Protein %	*DGAT1* p.K232A	AA - KK	0.000476428	0.000162726
305-day average Protein %	*DGAT1* p.K232A	KA - KK	0.826301438	0.556574309
305-day average Protein %	*PPP1R16A* rs109146371	CC - CT	7.04E-01	0.424915735
305-day average Protein %	*PPP1R16A* rs109146371	CC - TT	4.51E-04	1.54E-04
305-day average Protein %	*PPP1R16A* rs109146371	CT - TT	1.94E-09	6.47E-10
305-day average SNF %	*DGAT1* p.K232A	AA - KA	2.72588E-10	9.08384E-11
305-day average SNF %	*DGAT1* p.K232A	AA - KK	0.000955962	0.000328698
305-day average SNF %	*DGAT1* p.K232A	KA - KK	0.747004334	0.467163836
305-day average SNF %	*PPP1R16A* rs109146371	CC - CT	8.49E-01	0.585801062
305-day average SNF %	*PPP1R16A* rs109146371	CC - TT	0.002612358	9.10E-04
305-day average SNF %	*PPP1R16A* rs109146371	CT - TT	2.83E-08	9.45E-09

Note: Tukey's HSD and unadjusted t-test *p*-values are shown for all genotype pairwise comparisons for each candidate SNP and 305-day trait. Tukey's HSD *p*-values account for multiple comparisons, whereas t-test *p*-values are unadjusted.
